# Acute kidney injury after infant cardiac surgery: a comparison of pRIFLE, KDIGO, and pROCK definitions

**DOI:** 10.1186/s12882-023-03306-y

**Published:** 2023-08-24

**Authors:** Peng Gao, Wang He, Yu Jin, Chun Zhou, Peiyao Zhang, Wenting Wang, Jinxiao Hu, Jinping Liu

**Affiliations:** 1 Department of Anesthesiology, Peking Union Medical College Hospital, Chinese Academy of Medical Sciences and Peking Union Medical College, Beijing, China; 2 Pediatric Cardiac Surgery Center, Fuwai Hospital, National Center for Cardiovascular Diseases, Chinese Academy of Medical Sciences and Peking Union Medical College, Fuwai Hospital, No.167, North Lishi Road, Xicheng District, Beijing, China

**Keywords:** Acute kidney injury, Pediatric cardiac surgery, Infants, KDIGO, pROCK, pRIFLE

## Abstract

**Background:**

KDIGO and pRIFLE classifications are commonly used in pediatric acute kidney injury (AKI). As a novel AKI definition, pROCK considered the high variability of serum creatinine in children. This study aimed to compare the above three definitions for AKI in infants undergoing cardiac surgery.

**Methods:**

We analyzed a clinical cohort of 413 infants undergoing cardiac surgery. AKI was defined and staged according to pRIFLE, KDIGO, and pROCK, respectively. Incidence differences and diagnostic agreement across definitions were assessed. The association between postoperative outcomes and AKI by each definition was investigated.

**Results:**

Postoperative AKI was identified in 185 (44.8%), 160 (38.7%), and 77 (18.6%) patients according to pRIFLE, KDIGO, and pROCK, respectively. The agreement between pRIFLE and KDIGO was almost perfect (κ = 0.88), while there was only a slight agreement between pROCK and them. AKI by pROCK was independently associated with adverse outcomes (*p* = 0.003) and prolonged mechanical ventilation (*p* = 0.002).

**Conclusions:**

There were considerable differences in AKI incidence and staging among definitions. Compared with pRIFLE and KDIGO, AKI defined by pROCK was significantly reduced and better associated with postoperative adverse outcomes.

**Supplementary Information:**

The online version contains supplementary material available at 10.1186/s12882-023-03306-y.

## Background

Acute kidney injury (AKI) is a common finding after pediatric cardiac surgery, especially in young infants [1, 2]. It is also associated with increased mortality and morbidity [1, 3]. There have been many AKI definitions, which has made it difficult to compare results across studies. In 2004, the Acute Dialysis Quality Initiative group proposed a definition for AKI: the Risk, Injury, Failure, Loss of Kidney Function, and End-stage Kidney Disease (RIFLE) definition, which was the first evidence-based consensus [4]. Since then, in 2007, RIFLE criteria were modified into pediatric RIFLE (pRIFLE) to adapt the application in children, and pRIFLE was suggested to characterize the pattern of AKI in children [5]. Later, a new classification was introduced by the Kidney Disease: Improving Global Outcomes (KDIGO) Acute Kidney Injury Work Group in 2012 [6]. This classification included three stages of AKI according to relative changes in serum creatinine (SCr) and urine output. The above definitions have been evaluated in many studies of pediatric patients with AKI and showed good predictive ability for adverse outcomes [7–9].

Recently, the latest criterion for AKI in children was proposed, which was based on the reference change value (RCV) of SCr, called pediatric reference change value optimized for AKI in children (pROCK) [10]. Due to the high variability of serum creatinine in children, pRIFLE and KDIGO criteria might lead to overdiagnosis of AKI, and pROCK criterion could improve the detection of "true" AKI [10]. However, there is no report about the application of pROCK criterion as AKI definition in infants undergoing cardiac surgery. The objective of this study was to investigate the incidence of AKI in infants undergoing cardiac surgery with cardiopulmonary bypass (CPB) according to pRIFLE, KDIGO, and pROCK criteria, and to assess the association with in-hospital adverse outcomes.

## Materials and methods

### Study design

This study was a secondary analysis of the database of a prospective observational study in infants undergoing elective cardiac surgery with CPB [11]. The purpose of the original study was to investigate the association between oxygen delivery during CPB and postoperative AKI. Low-weight infants (≤ 10 kg) undergoing cardiac surgery with an expected CPB duration of 1–3 h qualified for the study. From August 2021 to July 2022, a total of 451 infants were screened for eligibility, and 38 were excluded. The exclusion criteria were patients with preoperative complications, previous cardiac surgery, and expected circulatory arrest during CPB. Only the first operation that each patient underwent was considered for the present analysis.

The study has been approved by the Medical Ethics committee of Fuwai Hospital, Chinese Academy of Medical Sciences and Peking Union Medical College. Written informed consent was obtained from the parents of the participants during the original study. All methods were carried out in accordance with relevant guidelines and regulations or declaration of Helsinki. This study was reported as per the STROBE guidelines.

### Clinical practice

General anesthesia was performed under the institutional standard protocol. The standard CPB circuit consisted of a roller pump (Stockert S5, Sorin, Germany), a hollow-fiber membrane oxygenator (Fx05, Terumo, Japan), and a hemofilter (Maquet BC20, Hirrlingen, Germany). Modified St. Thomas solution or histidine-tryptophan-ketoglutarate solution was used for cold cardioplegia in all patients according to expected CPB duration. Patients were rewarming to the nasopharyngeal temperature above 36 °C with a satisfactory hemodynamic state before weaning from CPB, and modified ultrafiltration was routinely performed. After the operation, all patients were transferred to the pediatric intensive care unit (PICU) and received postoperative treatment offered by a fixed pediatric cardiac team.

### Data collection

Demographic characteristics, intraoperative data, perioperative laboratory tests including routine blood and biochemistry examination, postoperative duration of mechanical ventilation (MV), PICU length of stay (LOS), and hospital LOS were recorded. The cardiac surgical procedures were graded as classes 1 to 6 according to the complexity of the operation using the Risk Adjustment for Congenital Heart Surgery (RACHS) category.

### Definitions

Postoperative AKI was defined according to pRIFLE, KDIGO, and pROCK, respectively. For the diagnosis of AKI, only the SCr criteria were used. Urine output criteria were not applied for the urine output could be influenced by many factors and data of precise urine were not available from our database. pRIFLE divided AKI into three severity stages (risk, injury, and failure) and two outcomes (loss and end-stage kidney disease), whereas both KDIGO and pROCK only had three severity stages (stage 1, stage 2, and stage 3). For the sake of clarity, we adopted stages 1–3 correspond to risk, injury, and failure in pRIFLE, respectively. Stage-specific definitions of the three classifications were provided in Supplementary Table 1. For pRIFLE, the estimated glomerular filtration rate (eGFR) was calculated using the Schwartz formula [12], and AKI was defined by a ≥ 25% decrease in estimated creatinine clearance within 7 days [4]. KDIGO defined AKI as a relative increase of ≥ 50% in SCr within 7 days or an absolute increase of over 26.5 mmol/L within 2 days [6]. A relative increase of ≥ 30% combined with an absolute increase of ≥ 20 mmol/L in SCr within 7 days was used to define AKI according to pROCK [10]. In terms of AKI stages, pRIFLE classified AKI stages 2 and 3 as 50% and 75% decreases in estimated creatinine clearance, and for KDIGO were SCr increases of ≥ 100% and ≥ 200%, respectively. Whereas pROCK classified AKI stages 2 and 3 as SCr increases of ≥ 40 mmol/L & ≥ 60% and ≥ 80 mmol/L & ≥ 120%, respectively.

Baseline SCr was defined as the most recent value before surgery, which was generally obtained 1–3 days prior to surgery. Postoperative SCr used for AKI definition was the highest value in the 7 days after surgery. The analytical assay for creatinine determination was performed in the hospital clinical laboratory using enzymatic technique. The adverse outcome was defined as a composite of in-hospital outcomes including peritoneal dialysis, re-operation, re-intubation, pulmonary infection, extracorporeal membrane oxygenation (ECMO), and death. Patients were regarded to have a composite outcome when they underwent at least one of the above postoperatively. Additionally, prolonged MV was defined as > 50 h, and prolonged PICU stay was defined as > 10 days (both were the 90th percentile of the overall patients).

### Statistical analysis

Categorical variables were presented as frequencies with percentages. Continuous variables were expressed as mean ± standard deviation, or median with interquartile ranges (IQR) 25–75th percentile. The normal distribution of variables was assessed visually through Q-Q diagram and histogram. The Chi-square test or Fisher’s exact test was used for categorical variables as appropriate. T-test or Mann–Whitney U test was used for continuous variables according to the distribution. The agreement between the three definitions was assessed using Cohen’s kappa statistic. Interpretation of kappa (κ) values is as follows: 0.00–0.20, slight agreement; 0.21–0.40, fair agreement; 0.41–0.60, moderate agreement; 0.61–0.80, substantial agreement; and > 0.8, almost perfect agreement [13]. Additionally, a subgroup analysis of the incidence of AKI across the three definitions was conducted in patients with low levels of baseline SCr (≤ 30 umol/L).

To compare MV duration and PICU LOS in AKI groups according to the three definitions, Kaplan–Meier curves were plotted with the endpoints of extubation and PICU discharge, respectively. Patients without AKI in the corresponding definition were used as the reference group. Multivariable logistic regression was used to assess the association of each pRIFLE, KDIGO, and pROCK definition with adverse outcomes, adjusting for age, weight, CPB duration, and RACHS category.

For all the statistical tests, a *p*-value < 0.05 was considered significant. The data were analyzed using statistical software SPSS 25.0 (SPSS Inc., Chicago, IL, USA) and R 4.1.0 (The R Foundation for Statistical Computing, Vienna, Austria).

## Results

### Patient population

**Fig. 1:**
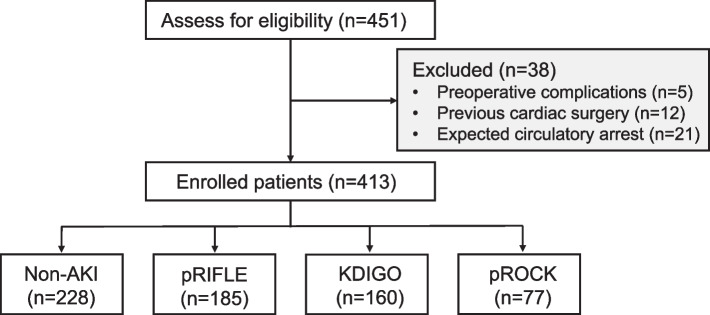
Flow chart of the study. “Non-AKI” refers to the patients defined as non-AKI by all the three diagnostic criteria

From August 2021 to July 2022, a total of 413 patients were consecutively enrolled in the original study. 38 patients were excluded: 5 had complications before surgery, 12 received previous cardiac surgery, and 21 for an expected circulatory arrest during CPB (Fig. 1). For the patients included, the median age was 195 days (IQR 130–288 days) with a median weight of 6.9 kg (IQR 5.7–8.2 kg). All the patients underwent CPB and aortic cross-clamping (ACC) with a duration of 83 min (IQR 64–106 min) and 54 min (IQR 42–77 min), respectively. The types of cardiac surgery were: atrial septal defect 23 (5.6%), ventricular septal defect 205 (49.6%), Tetralogy of Fallot 66 (16.0%), mitral/aortic valvuloplasty 56 (13.6%), pulmonary stenosis 18 (4.4%), total anomalous pulmonary venous connection 16 (3.9%), total endocardial cushion defect 14 (3.4%), double outlet right ventricle12 (2.9%), and pulmonary atresia 3 (0.7%).

According to the three AKI definitions, patients were assigned into four groups to show baseline characteristics: non-AKI by the three definitions, AKI by pRIFLE, AKI by KDIGO, and AKI by pROCK (Table 1). The patients with AKI identified by all three criteria were younger and with lower baseline hemoglobin and creatinine (*p* < 0.05). AKI patients also had lower white blood cells and higher direct bilirubin (*p* < 0.05).

**Table 1:** Demographic characteristics and operative data

Variables	Non-AKI (*n* = 228)	AKI by pRIFLE (*n* = 185)	AKI by KDIGO (*n* = 160)	AKI by pROCK (*n* = 77)
Age (day)	202 (139–311)	178 (122–258)	182 (121–260)	156 (105–237)
Male gender	118 (51.8%)	95 (51.4%)	82 (51.2%)	44 (57.1%)
RACHS category ≥ 3	39 (17.1%)	32 (17.3%)	29 (18.1%)	13 (16.9%)
2003Weight (kg)	7.06 ± 1.60	6.81 ± 1.68	6.77 ± 1.70	6.47 ± 1.76
Body length (cm)	67.73 ± 7.23	65.95 ± 7.58	65.86 ± 7.72	64.13 ± 7.67
Premature delivery	27 (11.8%)	21 (11.4%)	21 (13.1%)	12 (15.6%)
Birth weight (kg)	3.14 ± 0.59	3.14 ± 0.64	3.11 ± 0.65	3.10 ± 0.69
PAH	103 (45.2%)	82 (44.3%)	72 (45.0%)	41 (53.2%)
LVEF (%)	70 (67–73)	70 (65–73)	69 (65–73)	70 (65–73)
LVEDD (mm)	26.72 ± 7.12	27.02 ± 6.17	26.81 ± 6.29	25.88 ± 6.88
WBC (10^9^/L)	9.42 ± 2.90	8.80 ± 2.46	8.79 ± 2.53	8.78 ± 2.46
Hemoglobin (g/L)	117.59 ± 20.02	112.90 ± 15.56	112.97 ± 16.13	113.66 ± 19.12
Albumin (g/L)	42.02 ± 3.30	41.90 ± 3.15	41.98 ± 3.26	41.69 ± 3.59
AST (IU/L)	23 (18–34)	23 (17–38)	23 (17–37)	24 (17–37)
ALT (IU/L)	51 (43–61)	49 (39–62)	48 (38–61)	47 (38–59)
TBIL (umol/L)	5.85 (3.96–8.65)	6.50 (4.49–9.30)	6.81 (4.34–9.82)	6.75 (4.76–12.05)
DBIL (umol/L)	1.43 (0.92–2.38)	1.80 (1.18–2.76)	1.93 (1.21–3.11)	2.35 (1.40–3.46)
SCr (umol/L)	29.75 ± 8.48	23.96 ± 6.29	23.52 ± 6.19	23.67 ± 6.42
Cystatin-C (mg/L)	1.12 ± 0.24	1.11 ± 0.21	1.10 ± 0.21	1.14 ± 0.21
BUN (umol/L)	3.35 (2.28–4.64)	2.86 (2.11–4.36)	2.98 (2.16–4.48)	3.14 (2.14–4.58)
CPB duration (min)	83 (66–106)	85 (61–106)	83 (61–105)	90 (71–118)
ACC duration (min)	54 (43–77)	55 (41–76)	54 (41–76)	56 (45–80)
Nadir temperature (℃)	30.79 ± 1.48	30.97 ± 1.46	31.01 ± 1.51	30.86 ± 1.73

*AKI* Acute kidney injury, *pRIFLE* Pediatric-modified Risk, Injury, Failure, Loss, and End-Stage, *KDIGO* Kidney Disease: Improving Global Outcomes, *pROCK* pediatric reference change value optimized for AKI in children, *RACHS* Risk Adjustment for Congenital Heart Surgery, *PAH* Pulmonary arterial hypertension, *LVEF* left ventricular ejection fraction, *LVEDD* left ventricular end-diastolic dimension, *WBC* White blood cell, *TBIL* Total bilirubin; *DBIL* direct bilirubin, *AST* Aspartate aminotransferase, *ALT* Alanine aminotransferase, SCr, serum creatinine, *BUN* Blood urea nitrogen (umol/L), *CPB* Cardiopulmonary bypass, *ACC* Aortic cross-clamping

### AKI incidence and agreement between definitions

In the study population of 413 patients, 185 (44.8%) had AKI according to pRIFLE, 160 (38.7%) according to KDIGO, and 77 (18.6%) according to pROCK (Table 2). The incidences of overall AKI were different between the three definitions (*p* < 0.001). The incidences of stage 1 AKI according to pRIFLE, KDIGO, and pROCK were 33.9%, 26.2%, and 17.9% respectively; and the incidences of stage 2 AKI were 10.9%, 10.2%, and 0.7%, respectively. 10 (2.4%) patients were identified as stage 3 AKI by KDIGO, and no patient was stage 3 AKI according to pRIFLE and pROCK criteria.

**Table 2:** Agreement between pRIFLE, KDIGO, and pROCK classifications

Definition	**pROCK**			
	Non-AKI *n* = 336	Stage 1 *n* = 74	Stage 2 *n* = 3	Total *n* = 413
**KDIGO**				
Non-AKI	253 (75.3%)	0	0	253 (61.3%)
Stage 1	76 (22.6%)	32 (43.2%)	0	108 (26.2%)
Stage 2	7 (2.1%)	33 (44.6%)	2 (66.7%)	42 (10.2%)
Stage 3	0	9 (12.2%)	1 (33.3%)	10 (2.4%)
**pRIFLE**				
Non-AKI	228 (67.9%)	0	0	228 (55.2%)
Risk	102 (30.4%)	38 (51.4%)	0	140 (33.9%)
Injury	6 (1.8%)	36 (48.6%)	3 (100%)	45 (10.9%)

*AKI* Acute kidney injury, *pRIFLE* Pediatric-modified Risk, Injury, Failure, Loss, and End-Stage, *KDIGO* Kidney Disease: Improving Global Outcomes, *pROCK* Pediatric reference change value optimized for AKI in children

**Fig. 2:**
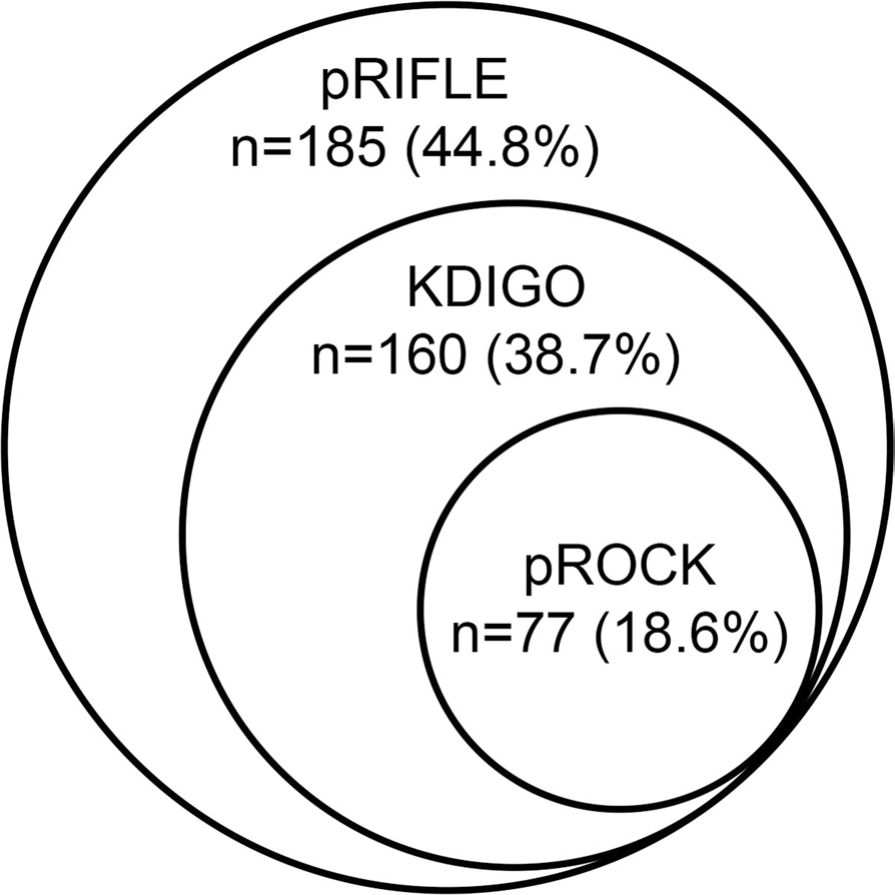
Definitional overlap of AKI according to the three definitions

AKI overlap across the three definitions was shown in Fig. 2. AKI patients diagnosed by pRIFLE covered all patients with AKI identified by KDIGO and pROCK. A total of 25 (6.1%) patients were diagnosed with AKI only by pRIFLE. According to pROCK, 58.37% (108/185) of the pRIFLE-AKI patients were reclassify as non-AKI, and 51.87% (83/160) of the KDIGO-AKI patients were reclassify as non-AKI.

The three definitions did not lead to a similar diagnosis or staging of AKI. The agreement between pRIFLE and KDIGO was almost perfect, while there was only a slight agreement between pROCK and them. Regarding the diagnosis of AKI, pRIFLE agreed KDIGO with 93.9% (κ = 0.88) of the time, pRIFLE agreed pROCK with 73.8% (κ = 0.44) of the time, and KDIGO agreed pROCK with 79.9% (κ = 0.53) of the time. Additionally, patients with AKI were staged differently among the three definitions. pRIFLE and KDIGO agreed on AKI stage 89.8% (κ = 0.82) of the time, pRIFLE and pROCK agreed on AKI stage 65.1% (κ = 0.29) of the time, and KDIGO and pROCK agreed on AKI stage 69.5% (κ = 0.33) of the time.

Moreover, as shown in Supplementary table 2, in the 274 (66.3%) patients with baseline creatinine ≤ 30 umol/L, a higher percentage of AKI was identified by all three definitions (*p* < 0.001). And the incidence of AKI was significantly higher according to pRIFLE and KDIGO compared with pROCK (55.5% and 49.6% vs 23.7%, *p* < 0.001). The difference in AKI incidence between patients with baseline SCr ≤ 30 umol/L and > 30 umol/L was over 30% according to pRIFLE and KDIGO, while it was 15.1% for pROCK.

### Comparison of clinical outcomes

Among the 413 patients included, postoperative composite morbidity was 7.5%. As shown in Table 3, the incidence of composite outcome was higher in patients with AKI according to pROCK classification (16.9% vs 5.4%, *p* = 0.001). However, there was no significant difference between patients with or without AKI according to the other two definitions (pRIFLE, 9.2% vs 6.1%, *p* = 0.242; KDIGO, 8.1% vs 7.1%, *p* = 0.704).

**Table 3:** Clinical outcomes

	pRIFLE		KDIGO		pROCK	
	Non-AKI (*n* = 228)	AKI (*n* = 185)	Non-AKI (*n* = 253)	AKI (*n* = 160)	Non-AKI (*n* = 336)	AKI (*n* = 77)
MV duration (hour)	11 (6–25)	19 (7–26.5)	**11 (6–25)**	19 (7–26.5)*	**11 (6–24)**	**24 (9–50)** †
Prolonged MV	22 (9.6%)	19 (10.3%)	24 (9.5%)	17 (10.6%)	**24 (7.1%)**	**17 (22.1%)**†
PICU LOS (day)	3.5 (2–5)	3 (1–5)	3 (2–5)	3 (2–5)	**3 (2–5)**	**5 (3–8)** †
Prolonged PICU stay	20 (8.8%)	21 (11.4%)	23 (9.1%)	18 (11.3%)	**27 (8.0%)**	14 (18.2%)**
Hospital LOS (day)	13 (10–17)	13 (10–17)	13 (10–17)	13 (10–17)	**13 (10–17)**	15 (11–21)**
**Composite outcome**	14 (6.1%)	17 (9.2%)	18 (7.1%)	13 (8.1%)	**18 (5.4%)**	13 (16.9%)**
Peritoneal dialysis	4 (1.8%)	3 (1.6%)	4 (1.6%)	3 (1.9%)	4 (1.2%)	3 (3.9%)
Re-operation	5 (2.2%)	4 (2.2%)	5 (2.0%)	4 (2.5%)	5 (1.5%)	4 (5.2%)
Re-intubation	4 (1.8%)	5 (2.7%)	6 (2.4%)	3 (1.9%)	6 (1.8%)	3 (3.9%)
Pulmonary infection	9 (3.9%)	13 (7.0%)	11 (4.3%)	11 (6.9%)	**11 (3.3%)**	**11 (14.3%)**†
ECMO	2 (0.9%)	1 (0.5%)	2 (0.8%)	1 (0.6%)	2 (0.6%)	1 (1.3%)
Mortality	0	1 (0.5%)	0	1 (0.6%)	0	1 (1.3%)

*AKI* Acute kidney injury, *pRIFLE* Pediatric-modified Risk, Injury, Failure, Loss, and End-Stage; *KDIGO* Kidney Disease: Improving Global Outcomes, *pROCK* Pediatric reference change value optimized for AKI in children, *MV* Mechanical ventilation, *PICU* Pediatric intensive care unit, *LOS* Length of stay, *ECMO* Extracorporeal membrane oxygenation

Bold values represent statistical significance, *: *p* < 0.05, **: *p* < 0.01, †: *p* < 0.001

MV duration was longer in patients with AKI according to KDIGO and pROCK, but showed no significant difference in pRIFLE (pRIFLE, *p* = 0.071; KDIGO, *p* = 0.048, pROCK, *p* < 0.001). In AKI patients according to pROCK, the incidence of prolonged MV was higher (22.1% vs 7.1%, *p* < 0.001). There was no difference in prolonged MV among patients with AKI versus non-AKI according to pRIFLE and KDIGO classification (Table 3).

In terms of postoperative PICU stay, the median LOS was longer in AKI patients according to pROCK, but not significant for pRIFLE and KDIGO (pRIFLE, 3.5 days [IQR 2–5 days] vs 3 days [IQR 1–5 days], *p* = 0.70; KDIGO, 3 days [IQR 2–5 days] vs 3 days [IQR 2–5 days], *p* = 0.842; pROCK, 3 days [IQR 2–5 days] vs 5 days [IQR 3–8 days], *p* = 0.001). The incidence of prolonged PICU stay was higher in patients identified as AKI by pROCK, (18.2% vs 8.0%, *p* = 0.007), but this was not significant in pRIFLE and KDIGO (Table 3).

**Fig. 3:**
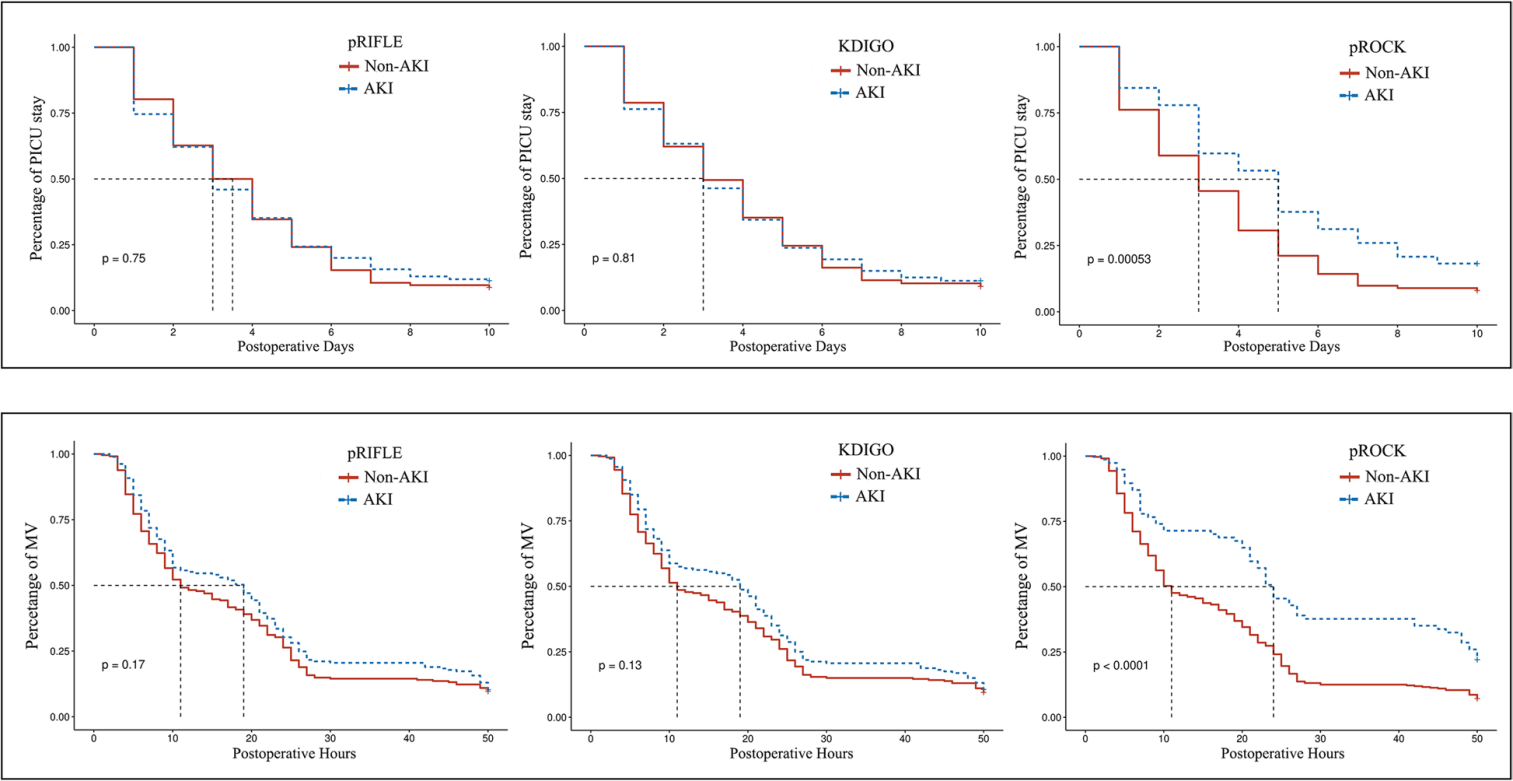
Comparison of postoperative PICU LOS and MV duration in AKI patients according to the three definitions

As shown in Fig. 3, pROCK criteria yielded a better separation (*p* < 0.001) between non-AKI and AKI patients on the Kaplan–Meier curves than pRIFLE and KDIGO in MV duration and PICU LOS. In multivariable logistic regression for adverse outcomes, after adjusting for age, weight, CPB duration, and RACHS category, AKI by pROCK was an independent risk factor for the composite outcome (OR 3.293, 95%CI 1.487–7.292, *p* = 0.003) and prolonged MV (OR 3.211, 95%CI 1.530–6.738, *p* = 0.002). Although AKI by pROCK was associated with prolonged PICU stay in univariable logistic regression, it was not significant in multivariate analysis (*p* = 0.118). Additionally, AKI by pRIFLE and KDIGO was not associated with in-hospital adverse outcomes (Table 4).

**Table 4:** Multivariable logistic regression for adverse outcomes

Adverse Outcomes	OR	95% CI	*P*
**Composite outcome**			
AKI by pRIFLE	1.643	0.769–3.509	0.20
AKI by KDIGO	1.172	0.546–2.512	0.684
AKI by pROCK	3.293	1.487–7.292	0.003
**Prolonged MV**			
AKI by pRIFLE	1.132	0.565–2.271	0.726
AKI by KDIGO	1.179	0.583–2.385	0.646
AKI by pROCK	3.211	1.530–6.738	0.002
**Prolonged PICU stay**			
AKI by pRIFLE	1.257	0.617–2.559	0.529
AKI by KDIGO	1.121	0.545–2.307	0.757
AKI by pROCK	1.860	0.854–4.052	0.118

*AKI* Acute kidney injury, *pRIFLE* Pediatric-modified Risk, Injury, Failure, Loss, and End-Stage, *KDIGO* Kidney Disease: Improving Global Outcomes, *pROCK* Pediatric reference change value optimized for AKI in children, *MV* Mechanical ventilation, *PICU* Pediatric intensive care unit

## Discussion

In this study, we compared the incidence of postoperative AKI and the association with adverse outcomes, according to pRIFLE, KDIGO, and pROCK definitions. As far as we know, this was the first analysis using pROCK classification to investigate AKI after infant cardiac surgery. We found that there were considerable differences in AKI incidence and staging among definitions. Compared with pRIFLE and KDIGO, fewer patients were identified as AKI according to pROCK, especially for patients with low levels of baseline SCr. Moreover, AKI was independently associated with in-hospital adverse outcomes and prolonged MV, for the patients diagnosed by pROCK but not by RIFLE or KDIGO.

AKI was a common finding after pediatric cardiac surgery [1, 2]. Even mild AKI was associated with increased morbidity and mortality [14, 15]. Several criteria have been described to define AKI in children, among which pRIFLE and KDIGO were the most commonly used in current practice [16]. Estimated creatinine clearance was used to define AKI according to pRIFLE [5], while KDIGO classification was based on the relative changes in SCr [6]. According to different definitions, the incidence of AKI after pediatric cardiac surgery varied greatly, ranging from 15 to 64% [17, 18]. A Meta-analysis reported the pooled incidence rate of AKI after pediatric cardiac surgery was 38.4% (95% CI 32.0%-44.7%) [19]. In our data, the incidence was 38.7% according to KDIGO and 44.8% according to pRIFLE, which was comparable to previous reports [20, 21]. However, the association between postoperative outcomes and AKI defined by pRIFLE and KDIGO was not detected like that in previous studies.

Recently, as a novel SCr-based definition for pediatric AKI, pROCK was developed from a large cohort of hospitalized children [10]. In pROCK definition, the RCV of SCr was estimated based on age and baseline SCr level, and AKI was defined as SCr increase over RCV of SCr. Neither pRIFLE nor KDIGO took into account the high variability of changes in SCr, which did not reflect a real change in renal function [22]. Xu et al. [10] indicated that a greater increase in SCr than normal variability might better represent the true decrease in renal function in children. In their study, 5.3%, 10.2%, and 15.2% of patients were identified as AKI according to pROCK, KDIGO, and pRIFLE, respectively [10]. About 66% of AKI patients defined by pRIFLE and 51% of AKI patients defined by KDIGO were reclassified as non-AKI by pROCK, and mortality risk in these children was comparable with those without AKI. Therefore, the investigators of pROCK concluded it improved the detection of “true” AKI in children compared with earlier definitions that might lead to overdiagnosis of AKI. Our data also showed the incidence of AKI was 18.6% according to pROCK, which was significantly lower than that defined by pRIFLE (44.8%) and KDIGO (38.7%).

Both KDIGO and pRIFLE defined AKI by a fixed percentage increase in SCr (eGFR used in pRIFLE was calculated from SCr), which would lead to limited accuracy of AKI diagnosis in patients with lower levels of baseline SCr. The relative changes seemed less reliable when the baseline SCr was low [23]. However, relatively low SCr was a common characteristic of young children [10]. In our patients, the mean baseline SCr was 27.15 ± 8.10 umol/L, and a total of 274 (66.3%) patients had baseline SCr ≤ 30 umol/L. The incidence of AKI identified by KDIGO and pRIFLE was significantly higher (approximately 50%) in children with baseline SCr ≤ 30 μmol/L. Among these AKI patients defined by pRIFLE or KDIGO, more than one-half of the AKI cases were reclassified as non-AKI by pROCK. Clinically, a relative increase in SCr of > 50% was common in patients with low baseline SCr, while an absolute increase of > 20 μmol/L would be obviously less. Thus, pROCK might avoid overdiagnosis of AKI, particularly in children with lower baseline SCr, and help in detecting “true” AKI in children. Our results also demonstrated that AKI defined by pROCK was more strongly associated with adverse outcomes and MV duration than AKI defined by the other two definitions.

As a novel definition for pediatric AKI, there was limited evidence on the application of pROCK criteria in pediatric cardiac surgery at present. Nevertheless, the high specificity of pROCK criteria was determined in critically ill children [24]. Moreover, in terms of the association with risk of mortality, pROCK was slightly stronger than that of KDIGO [25]. There might be the risk of overdiagnosis in current AKI definitions [26], as our results showed no differences in postoperative adverse outcomes between AKI and non-AKI patients according to pRIFLE and KDIGO. In contrast, our results were not compatible with many reports, in which AKI was regarded as an important indicator of mortality and health care costs in cardiac surgery [27, 28]. In addition, as the creator of pRIFLE, Stuart L. Goldstein [29] indicated that the use of definition with high specificity could lead to less attention to AKI patients who were ruled out by pROCK. And pROCK was deficient in identifying patients at risk for renal dysfunction compared with pRIFLE. Despite this concern, pROCK consistently outperformed the creatinine criteria of KDIGO and pRIFLE in predicting both survival time and survival status [10]. Hopefully, as the further exploration of the correlation of AKI defined by pROCK with short- and long-term renal function and postoperative outcomes, pROCK would be adopted as a standard AKI definition in pediatric cardiac surgery.

The study had some limitations. Firstly, due to the limited sample size, severe AKI (stage 2–3) was rare. Therefore, we did not compare the differences of postoperative outcomes in each AKI stage. Moreover, since the study design of the original study, some relevant data was missed, such as vasopressor need, fluid balance the outcome follow-up. The application of pROCK in pediatric cardiac surgery needs to be further explored, and future research should include larger patient populations, such as neonates, as well as children with higher risk for postoperative AKI.

The association between long-term outcomes and AKI according to the three definitions remained to be explored.

## Conclusions

There were considerable differences in AKI incidence and staging among definitions. Compared with pRIFLE and KDIGO, the incidence of AKI was significantly decreased according to pROCK based on the RCV of SCr. Postoperative AKI defined by pROCK was also better associated with adverse outcomes, suggesting that it might be the preferable method for diagnosing AKI in low-weight infants (≤ 10 kg) undergoing cardiac surgery. The application of pROCK might reduce overdiagnosis and thus provide promise to improve the diagnostic ability of AKI after pediatric cardiac surgery.

### Supplementary Information

**Additional file 1:** **Supplementary Table 1.** Staged diagnostic criteria for AKI. **Supplementary Table 2.** AKI incidence in patients with baseline SCr≤30 umol/L.

## Data Availability

The datasets used and/or analysed during the current study are available from the corresponding author on reasonable request.

## Abbreviations

AKI: Acute kidney injury
RIFLE: Risk, Injury, Failure, Loss of Kidney Function, and End-stage Kidney Disease
pRIFLE: Pediatric RIFLE
KDIGO: Kidney Disease: Improving Global Outcomes
Scr: Serum creatinine
RCV: Reference change value
pROCK: Pediatric reference change value optimized for AKI in children
CPB: Cardiopulmonary bypass
PICU: Pediatric intensive care unit
MV: Mechanical ventilation
LOS: Length of stay
RACHS: Risk Adjustment for Congenital Heart Surgery
eGFR: Estimated glomerular filtration rate
ECMO: Extracorporeal membrane oxygenation
IQR: Interquartile ranges
