# Supplementary material for: Acute kidney injury after infant cardiac surgery: a comparison of pRIFLE, KDIGO, and pROCK definitions
Source: BMC Nephrol. 2023 Aug 24;24:251. doi: 10.1186/s12882-023-03306-y (PMC10464137; doi:10.1186/s12882-023-03306-y)
Supplement: Supplementary file 1 — Additional file 1: Supplementary Table 1. Staged diagnostic criteria for AKI. Supplementary Table 2. AKI incidence in patients with baseline SCr≤30 umol/L. [file 12882_2023_3306_MOESM1_ESM.docx]

| Definition for AKI Stages |
| --- |
| **pRIFLE** |
| Stage 1 (Risk): eGFR decreased by 25% |
| Stage 2 (Injury): eGFR decreased by 50% |
| Stage 3 (Failure): eGFR decrease by 75% or eGFR < 35 ml/min per 1.73 m^2^ |
|  |
| **KDIGO** |
| Stage 1: Increase in creatinine of ≥ 50% or absolute increase in creatinine of 0.3 mg/dL |
| Stage 2: Increase in creatinine of ≥ 100% |
| Stage 3: Increase in creatinine of ≥ 200% or eGFR ≤ 35 ml/min per 1.73 m^2^ |
|  |
| **pROCK** |
| Stage 1: Increase in creatinine of both ≥ 30% and ≥ 20 umol/L |
| Stage 2: Increase in creatinine of both ≥ 60% and ≥ 40 umol/L |
| Stage 3: Increase in creatinine of both ≥ 120% and ≥ 80 umol/L |

**Supplementary Table 1.** Staged diagnostic criteria for AKI

AKI, acute kidney injury; pRIFLE, pediatric-modified Risk, Injury, Failure, Loss, and End-Stage; eGFR, estimated glomerular filtration rate; KDIGO, Kidney Disease: Improving Global Outcomes; pROCK, pediatric reference change value optimized for AKI in children.

**Supplementary Table 2.** AKI incidence in patients with baseline SCr≤30 umol/L

| Definitions | Baseline SCr ≤30 umol/L  n= 274 | Baseline SCr > 30 umol/L  n = 139 | *P* |
| --- | --- | --- | --- |
| AKI by pRIFLE | 152 (55.5%) | 33 (23.7%) | < 0.001 |
| AKI by KDIGO | 136 (49.6%) | 24 (17.3%) | < 0.001 |
| AKI by pROCK | 65 (23.7%) | 12 (8.6%) | < 0.001 |

AKI, acute kidney injury; pRIFLE, pediatric-modified Risk, Injury, Failure, Loss, and End-Stage; KDIGO, Kidney Disease: Improving Global Outcomes; pROCK, pediatric reference change value optimized for AKI in children; SCr, serum creatinine.
